# Cathelicidin hCAP18/LL-37 promotes cell proliferation and suppresses antitumor activity of 1,25(OH)_2_D_3_ in hepatocellular carcinoma

**DOI:** 10.1038/s41420-022-00816-w

**Published:** 2022-01-17

**Authors:** Huidan Zhang, Junai Zhen, Rong Zhang, Yangke Wanyan, Kehang Liu, Xueli Yuan, Liping Tao, Yuqing Chen

**Affiliations:** Jiangsu Province Key Laboratory for Molecular and Medical Biotechnology, Life Sciences College, Nanjing Normal University, Nanjing, China

**Keywords:** Oncogenes, Prognostic markers, RNAi

## Abstract

Cathelicidin hCAP18/LL-37 can resist infection from various pathogens and is an essential component of the human immune system. Accumulating evidence has indicated that hCAP18/LL-37 plays a tissue-specific role in human cancer. However, its function in hepatocellular carcinoma (HCC) is poorly understood. The present study investigated the effects of hCAP18/LL-37 on HCC in vitro and in vivo. Results showed that hCAP18/LL-37 overexpression significantly promoted the proliferation of cultured HCC cells and the growth of PLC/PRF-5 xenograft tumor. Transcriptome sequencing analyses revealed that the PI3K/Akt pathway was the most significant upregulated pathway induced by LL-37 overexpression. Further analysis demonstrated that hCAP18/LL-37 stimulated the phosphorylation of EGFR/HER2 and activated the PI3K/Akt pathway in HCC cells. Furthermore, stronger EGFR/HER2/Akt signals were observed in the PLC/PRF-5^LL-37^ xenograft tumor. Interestingly, even though the expression of hCAP18/LL-37 was significantly downregulated in HCC cells and tumors, 1,25(OH)_2_D_3_ treatment significantly upregulated the hCAP18/LL-37 level both in HCC cells and xenograft tumors. Moreover, 1,25(OH)_2_D_3_ together with si-LL-37 significantly enhanced the antitumor activity of 1,25(OH)_2_D_3_ in the PLC/PRF-5 xenograft tumor. Collectively, these data suggest that hCAP18/LL-37 promotes HCC cells proliferation through stimulation of the EGFR/HER2/Akt signals and appears to suppress the antitumor activity of 1,25(OH)_2_D_3_ in HCC xenograft tumor. This implies that hCAP18/LL-37 may be an important target when aiming to improve the antitumor activity of 1,25(OH)_2_D_3_ supplementation therapy in HCC.

## Introduction

Primary liver cancer is the sixth most commonly diagnosed cancer and the third leading cause of cancer death worldwide in 2020, with ~906,000 new cases and 830,000 deaths [1]. Hepatocellular carcinoma (HCC) originates in the hepatocyte epithelium, accounts for 75–85% of primary liver cancer, and represents a classic paradigm of inflammation-linked cancer [2]. In most cases, chronic liver inflammation and the resultant cirrhotic microenvironment stimulate the initiation and progression of HCC by promoting proliferative and survival signaling, as well as inducing genomic instability [2]. In chronic inflammatory environments, cytokines, growth factors, chemokines, and metabolites modulate the progression of HCC [3]. Despite recent improvements in therapeutic options for HCC, many conventional therapies, including surgical resection, transplantation, radiotherapy, local radiofrequency ablation, chemotherapy, and interventional therapies, are not satisfactory [4]. Approaches that utilize immunotherapies alone or in combination with molecularly targeted therapies are currently considered required tools for precision medicine-based treatment of HCC, particularly for advanced-stage HCC [5]. Hence, improved understanding of the molecular HCC mechanisms will contribute greatly to the development of new targeted or combination therapies.

Cathelicidin hCAP18/LL-37 is an essential component of the innate immune system against pathogen infection in humans, including several types of bacterial and viral infections [6]. It is synthesized from the precursor protein pre-hCAP18 (19 kDa) into propeptide hCAP18 (16 kDa) by excising the signaling peptide and is then processed by proteolytic cleavage into bioactive cathelicidin LL-37 (4.5 kDa) by specific serine proteases, such as proteinase 3 (PR3) [7]. LL-37 also exhibits diverse and tissue-specific immunomodulatory activities by activating pro- or anti-inflammatory mediators [8]. Similarly, accumulating evidence has indicated that LL-37 plays a dual role in human cancer, exerting either pro-tumorigenic or anticancer effects in different tumors [9]. LL-37 induces tumorigenic effects in several types of tumors, such as ovarian, lung, breast, prostate, pancreatic, malignant melanoma, and skin squamous cell carcinoma [10, 11]. The anticancer effect induced by LL-37 has also been observed in colon and gastric cancers, and oral squamous cell carcinoma [12]. Different membrane receptors in various cancer cells appear to be responsible for tissue-specific or cell-specific effects induced by LL-37. Extensive LL-37 research conducted in breast, colon, lung, and gastric cancers revealed multiple mechanisms [13], although little is known about HCC.

Increasing interest in studying the role of vitamin D in cancer has been provided by the scientific literature during the last years. Several epidemiological investigations have shown that vitamin D (active form: 1,25(OH)_2_D_3_) deficiency or insufficiency is common not only in patients with chronic liver diseases but also in HCC patients [14–16]. Experimental studies have also revealed vitamin D antitumor effects in HCC, implying that vitamin D and its analogs may provide new treatment strategies for HCC patients [17, 18]. Thus, thorough understanding of the effect of vitamin D on HCC is necessary. *CAMP* gene (encoding pre-hCAP18) is an important primary vitamin D target gene in the VDR pathway, and hCAP18/LL-37 is induced by 1,25(OH)_2_D_3_ in several cell types, including various immune, epithelial, and some cancer cell [19, 20]. A prior report has shown that vitamin D can up-regulate peritoneal macrophage LL-37 expression, which results in enhanced immunological defense against spontaneous bacterial peritonitis in patients with cirrhosis and ascites [21]. However, whether anticancer activity of vitamin D is affected by hCAP18/LL-37 in HCC is still unknown.

The aim of the present study was to determine the function of hCAP18/LL-37 in human HCC utilizing in vitro and in vivo functional assays. Results demonstrated that hCAP18/LL-37 promotes tumor growth mainly by activating the EGFR/HER2/Akt signaling pathway in HCC cells and in xenograft tumors with endogenous overexpression. In addition, current results indicated that hCAP18/LL-37 was an important peptide that suppressed the antitumor activity of vitamin D on HCC xenografts.

## Results

### Expression of *CAMP* gene is decreased in human HCC tumor and cultured HCC cells

**Fig. 1:**
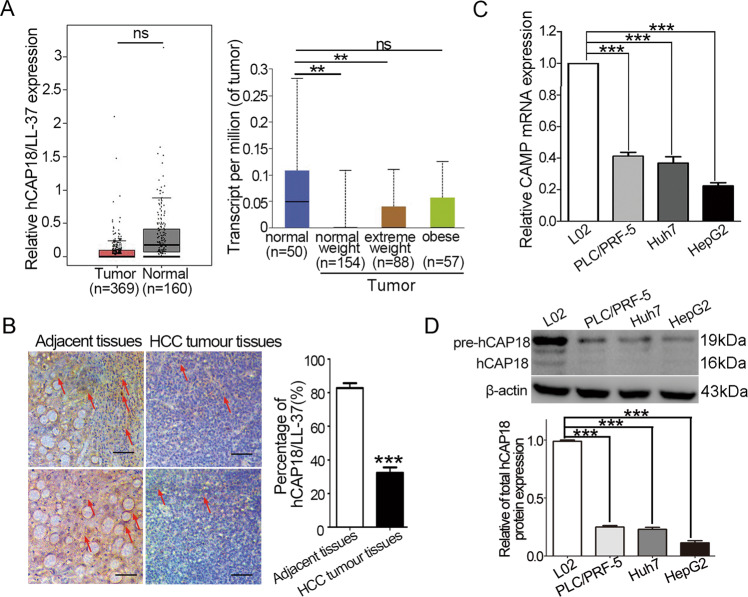
hCAP18/LL-37 expression levels in HCC tumors and cell lines. **A** *CAMP* mRNA levels in HCC tumor and normal liver tissues based on GEPIA and UALCAN databases. **B** Immunohistochemistry (IHC) shows the percentage of hCAP18/LL-37—positive cells (*n* = 60). Representative images of IHC staining analysis of hCAP18/LL-37 expression in HCC tissues and adjacent non-tumor tissues. The arrows indicate the hCAP18/LL-37 positive cells. Original magnification, ×200. Scale bars = 50 μm. **C** *CAMP* mRNA levels in HCC and normal L02 cells were detected by qRT-PCR. **D** pre-hCAP18 and hCAP18 levels in HCC and L02 cells were detected by western blotting using hCAP18/LL-37 antibody. Relative protein expression of total hCAP18 (per-hCAP18 and hCAP18) vs. β-actin was determined using ImageJ densitometry analysis. **p < 0.01, ***p < 0.001, and ns (no significance).

Using the GEPIA and UALCAN databases, the hCAP18 mRNA level was first investigated in HCC patients. A total of 160 normal individuals and 369 patients with HCC were included. The mRNA expression levels for *CAMP* gene were lower in HCC tumor tissues than in normal liver tissues (Fig. 1A). Further analysis revealed that *CAMP* mRNA level was significantly decreased in both normal weight and extreme-weight HCC patients compared to normal tissues (p < 0.001). However, there was no significant difference between tissues from obese HCC patients and normal tissues. Next, hCAP18/LL-37 levels were compared between 60 human HCC tissues and 60 paired adjacent normal tissues using tissue microarrays and immunohistochemistry (Fig. 1B). The hCAP18/LL-37 protein levels in HCC tissues were significantly lower than those in adjacent normal liver tissues (*p* < 0.001). Lastly, three HCC cell lines were chosen to check hCAP18/LL-37 basal levels (Fig. 1C, D). The *CAMP* mRNA levels were significantly lower in PLC/PRF-5, Huh7, and HepG2 cells compared to normal liver L02 cells (*p* < 0.001; Fig. 1C). Consistently, pre-hCAP18/hCAP18 protein levels in PLC/PRF-5, Huh7, and HepG2 cells were lower (Fig. 1D). Taken together, these data indicate that *CAMP* expression is downregulated in human HCC tumors and cultured HCC cells.

### LL-37 can promote HCC cells growth but leads to cell necrosis at very high concentrations

**Fig. 2:**
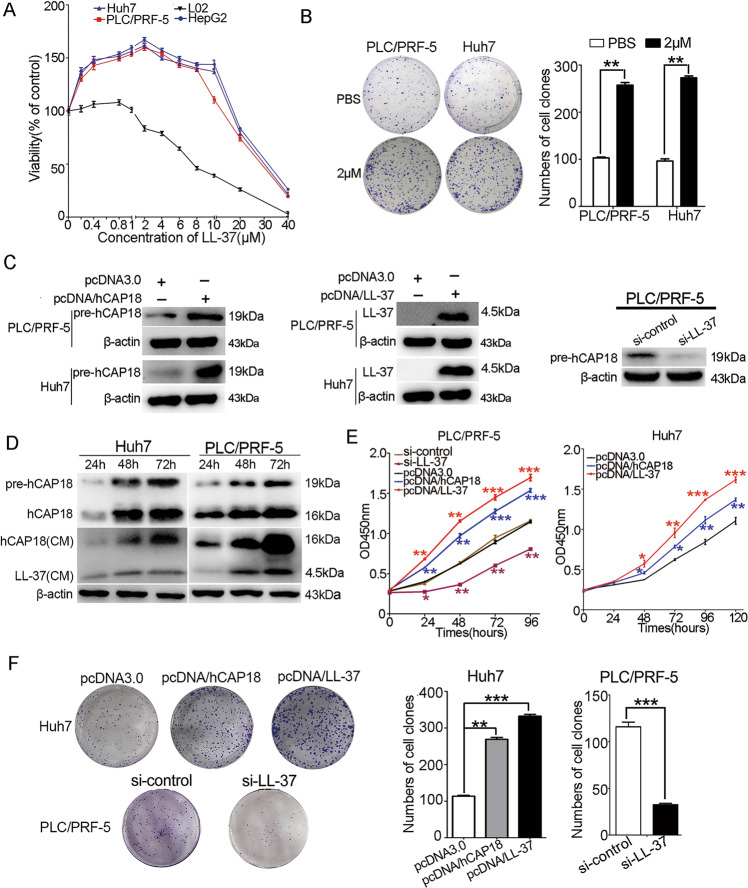
Effect of hCAP18/LL-37 on the HCC cells growth. **A** CCK-8 assay was conducted in PLC/PRF-5, Huh7, HepG2, and L02 cells after treatment with different concentrations (0–40 μM) of LL-37 for 24 h. **B** Effect of LL-37 (2 μM) on the colony formation detected by clone formation assays. **C** Huh7 and PLC/PRF-5 cells were transfected with pcDNA/hCAP18, pcDNA/LL-37, or si-LL-37 for 48 h, respectively. Efficiency of hCAP18/LL-37 overexpression or knockdown in PLC/PRF-5 and Huh7 cells was detected by western blotting. **D** Levels of pre-hCAP18 (~19 kDa), hCAP18 (~16 kDa), and LL-37 (~4.5 kDa) were detected after transfection with pcDNA/hCAP18 for 24 h, 48 h, 72 h, and 96 h. The effect on cell growth (**E**) and survival (**F**) were detected by CCK-8 assay and colony formation assay after transfection with pcDNA/hCAP18, pcDNA/LL-37, or si-LL-37. Data represent the mean ± SEM for 4–6 independent experiments. CM: culture medium. *p < 0.05, **p < 0.01, and ***p < 0.001.

To assess the effects of LL-37 on HCC cells, Huh7, HepG2, and PLC/PRF-5 cells were treated with LL-37 at different concentrations for 24 h. Cell viability was determined by an MTT assay (Fig. 2A). LL-37 promoted HCC cells growth in a certain concentration range. For Huh7, HepG2, and PLC/PRF-5 cells, the concentration for a maximum promoting effect was ~2 μM. The growth-promoting effect was also observed when the LL-37 concentration reached 10 μM. However, treatment with very high concentrations of LL-37 (such as 20 μM and 40 μM) induced necrosis in PLC/PRF-5, Huh7, and HepG2 cells, which was revealed by Hoechst 33342/PI staining (Supplementary Fig. S1A). Subsequently, clone formation assays were conducted in PLC/PRF-5 and Huh7 cells at LL-37 concentrations of 2 μM, which demonstrated that the colony formation ability was significantly increased after these treatments (*p* < 0.01; Fig. 2B).

Next, the effect of endogenous hCAP18/LL-37 expression on the viability and proliferation of Huh7 and PLC/PRF-5 cells was further investigated. Three vectors were designed, including a vector expressing precursor protein hCAP18 (pcDNA/hCAP18), LL-37 peptide (pcDNA/LL-37), and si-LL-37. The efficiency of hCAP18/LL-37 overexpression and knockdown was verified by western blotting (Fig. 2C). Since hCAP18/LL-37 is a secreted protein/peptide, hCAP18/LL-37 levels were detected in the culture medium after pcDNA/hCAP18 transfections with different durations. Results showed that both hCAP18 and LL-37 were detected in the culture medium, and the LL-37 level increased with transfection time (Fig. 2D). Then, CCK-8 and colony formation assays were used to determine the effect of hCAP18/LL-37 on viability and proliferation of HCC cells. The viability of both PLC/PRF-5 and Huh7 cells was significantly increased after hCAP18/LL-37 overexpression and significantly decreased after knockdown via si-LL-37 transfection (*p* < 0.01; Fig. 2E). Consistently, colony formation ability of PLC/PRF-5 and Huh7 cells was significantly enhanced after overexpression of hCAP18/LL-37, and significantly decreased after hCAP18/LL-37 knockdown (p < 0.01; Fig. 2F). Taken together, these data identified that hCAP18/LL-37 can promote HCC cell viability and growth in vitro.

### Expression of hCAP18/LL-37 in HCC cells promotes proliferation via cell cycle progression

**Fig. 3:**
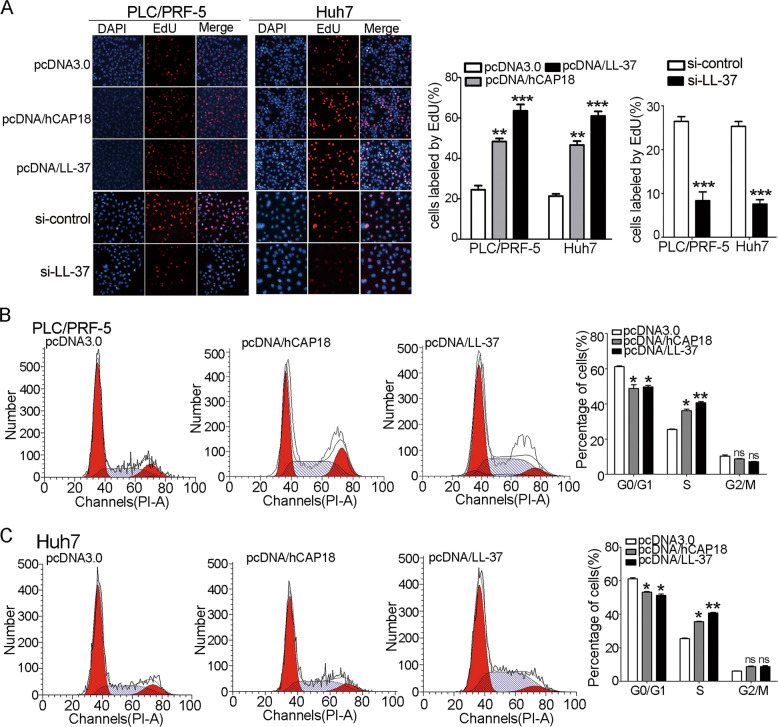
hCAP18/LL-37 expression promotes cell proliferation and cell cycle progression in HCC cells. **A** EdU proliferation assay was conducted in PLC/PRF-5 and Huh7 cells after transfection with pcDNA/hCAP18, pcDNA/LL-37, or si-LL-37, respectively. EdU-positive cells were counted for statistical analysis. Cell cycle analysis was performed in PLC/PRF-5 (**B**) and Huh7 (**C**) cells by PI staining and flow cytometry detection. The cells transfected with pcDNA3.0 were used as a control. Data represent the mean ± SEM for 4–6 independent experiments.*p < 0.05, **p < 0.01, ***p < 0.001, and ns (no significance).

To determine whether hCAP18/LL-37 affects HCC cells proliferation, EdU staining and cell cycle analysis were conducted. The overexpression of hCAP18/LL-37 in PLC/PRF-5 and Huh7 cells led to a significant enhancement of cell proliferation, as more EdU-positive cells were observed (*p* < 0.01; Fig. 3A). Significant proliferation inhibition was observed after si-LL-37 transfection. Cell cycle distribution in PLC/PRF-5 and Huh7 cells was analyzed using PI staining (Fig. 3B). Quantitative statistical data revealed that after transfection with pcDNA/hCAP18 or pcDNA/LL-37, cell population in the S phase was higher, and the proportion of cells in the G0/G1 phase was lower compared to the control group (Fig. 3C). Thus, overexpression of hCAP18/LL-37 significantly promoted the proliferation of PLC/PRF-5 and Huh7 cells by promoting cell cycle progression.

### hCAP18/LL-37 is involved in HB-EGF release and EGFR/HER2 activation in HCC cells

**Fig. 4:**
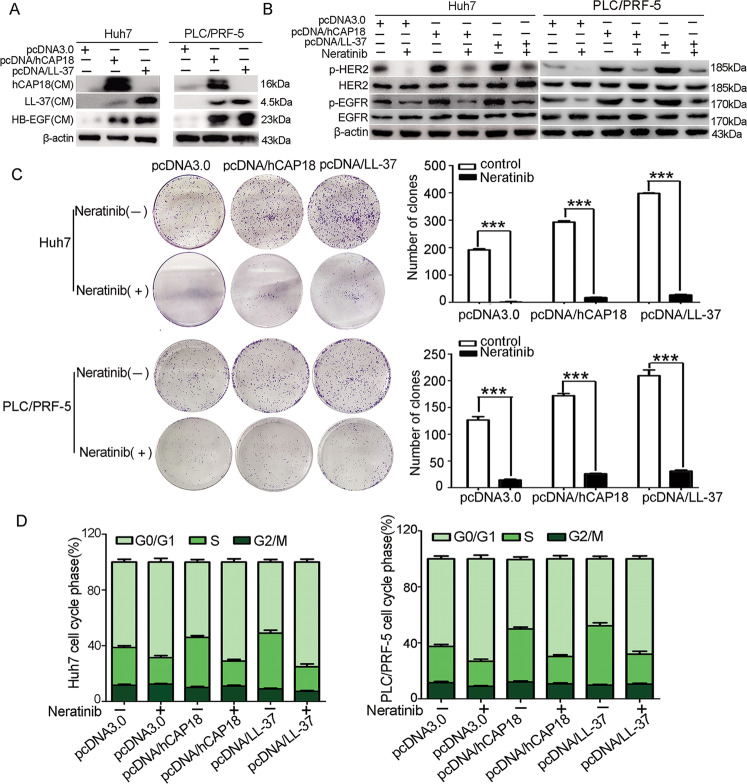
hCAP18/LL-37 expression induces HB-EGF release and activation of EGFR/HER2 in HCC cells. **A** Huh7 and PLC/PRF-5 cells were transfected with pcDNA/hCAP18 or pcDNA/LL-37 for 48 h. HB-EGF in culture medium (CM) was detected by western blotting. **B** Huh7 and PLC/PRF-5 cells were transfected with pcDNA/hCAP18 or pcDNA/LL-37 for 48 h, and then, western blot was conducted to detect the levels of p-EGFR (Tyr845) and p-HER2 (Tyr1248). Clone formation assay (**C**) and cell cycle assay (**D**) were conducted in Huh7 and PLC/PRF-5 cells with or without neratinib (3 μM) after pcDNA/hCAP18 or pcDNA/LL-37 transfection. For cell cycle assay, cell proportion changes during different cell cycle periods were analyzed using PI staining. Data are presented as the mean ± SEM for 4–6 different experiments. ***p < 0.001.

EGFR signaling pathway plays a significant role in tumor proliferation and angiogenesis in HCC pathogenesis [4]. A previous study has shown that LL-37 induces the release of HB-EGF, an EGFR family ligand, in keratinocytes and endothelial cells [22]. Here, HB-EGF was detected in Huh7 and PLC/PRF-5 cells after transfection with pcDNA/hCAP18 and pcDNA/LL-37 for 48 h (Fig. 4A). Almost no soluble HB-EGF was detected in the culture medium of control cells. However, overexpression of hCAP18/LL-37 in Huh7 and PLC/PRF-5 cells led to an obvious release of soluble HB-EGF into the culture medium, suggesting that hCAP18/LL-37 has the ability to induce the release of soluble HB-EGF from the membrane-bound forms. Since HB-EGF is the binding ligand of EGFR, further experiments were conducted to detect its effect on EGFR. It was found that hCAP18/LL-37 expression increased the phosphorylation level of EGFR (Tyr845) and HER2 (Tyr1248) in both Huh7 and PLC/PRF-5 cells, while p-EGFR (Tyr845) and p-HER2 (Tyr1248) induced by hCAP18/LL-37 was completely blocked by neratinib, an irreversible inhibitor target of EGFR and HER2 (Fig. 4B). Thus, hCAP18/LL-37 promoted the release of HB-EGF and activated the phosphorylation of EGFR and HER2. Consistently, the colony formation ability was significantly decreased after blocking the activation of EGFR and HER2 using neratinib in Huh7 and PLC/PRF-5 cells, which overexpressed hCAP18/LL-37 (*p* < 0.001; Fig. 4C). The effect of cell cycle promotion induced by hCAP18/LL-37 was also inhibited by neratinib, resulting from decreased populations of cells in the S phase after neratinib treatment together with hCAP18/LL-37 overexpression, compared to hCAP18/LL-37 overexpression alone (Fig. 4D). Taken together, these data indicate that hCAP18/LL-37 exerted its pro-proliferation effects in HCC cells partly via EGFR/HER2 activation.

### hCAP18/LL-37 exerts pro-proliferating effect mainly by activating PI3K/Akt signaling pathway in HCC cells

**Fig. 5:**
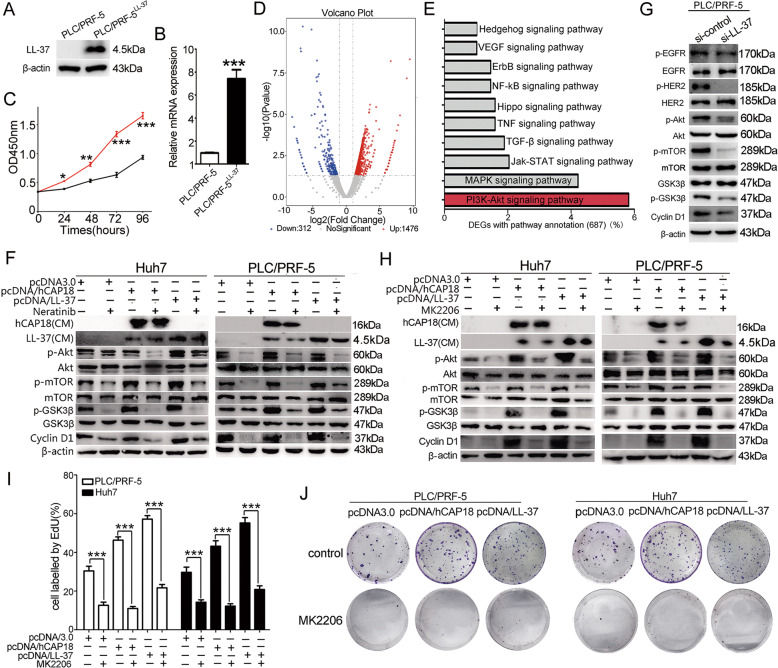
hCAP18/LL-37 regulates PI3K/Akt signaling pathway in HCC cells. Stable LL-37-overexpressing cell line PLC/PRF-5^LL-37^ was verified by western blotting (**A**), qRT-PCR (**B**), and cell growth curve analysis by CCK-8 assay (**C**). **D** Transcriptome sequencing identified genes involved in PLC/PRF-5^LL-37^. Volcano plot shows differential gene expression (red: upregulated; blue: downregulated) in PLC/PRF-5^LL-37^ cells compared to PLC/PRF-5 cells. Log2 fold change is represented on *x*-axis, while *y*-axis represents –log10 *p*-value. **E** Top ten KEGG pathways enriched in genes differentially expressed in PLC/PRF-5^LL-37^ cell. **F** PI3K/Akt pathway was measured in PLC/PRF-5 and Huh7 cells using western blotting. After transfection with pcDNA/hCAP18 or pcDNA/LL-37 for 24 h, 3 μM neratinib was added. Protein levels were detected another 24 h. **G** After transfection with si-LL-37 for 48 h, protein levels were measured in PLC/PRF-5 cells. **H** After pcDNA/hCAP18 or pcDNA/LL-37 transfection for 24 h, AKT inhibitor (MK2206, 5 μM) was added for another 24 h incubation and then protein levels were measured in PLC/PRF-5 and Huh7 cells. EdU proliferation assays (**I**) and colony formation assays (**J**) were conducted in Huh7 and PLC/PRF-5 cells with or without MK2206. Data are presented as the mean ± SEM for 4–6 different experiments. *p < 0.05, **p < 0.01, ***p < 0.001.

To explore the effect of hCAP18/LL-37 expression on cancer-related signaling pathways in HCC cells, whole transcriptome sequencing was performed using a stable LL-37-overexpressing cell line PLC/PRF-5^LL-37^. The LL-37 level and the growth curve of PLC/PRF-5^LL-37^ were identified at first (Fig. 5A–C). Differentially expressed genes in PLC/PRF-5^LL-37^ and PLC/PRF-5 cells were represented using volcano plots (Fig. 5D). There were 1788 differentially expressed genes (|log_2_fold change | >2, *p* < 0.05), which included 1476 upregulated and 312 downregulated genes in PLC/PRF-5^LL-37^ cells. KEGG pathway analysis showed that differential gene expression in PLC/PRF-5^LL-37^ cells was enriched in several oncogenic signaling pathways, such as PI3K/Akt, MAPK, and JAK/STAT (Fig. 5E). Among these, PI3K/Akt ranked first. Thus, LL-37 expression activated multiple oncogenic signaling pathways in PLC/PRF-5 cells, and the PI3K/Akt signaling pathway was the most significant upregulated pathway induced by LL-37 overexpression.

Next, the downstream signaling pathways of PI3K/Akt activation were further analyzed in HCC cell lines. It was found that hCAP18/LL-37 overexpression increased the expression level of p-Akt (Ser473), p-mTOR (Ser2448), and cyclin D1 in both Huh7 and PLC/PRF-5 cells (Fig. 5F). However, together with EGFR/HER2 inhibitor neratinib treatment, the enhancement signals above were decreased significantly (*p* < 0.001). Accordingly, LL-37 knockdown by si-LL-37 in PLC/PRF-5 cells resulted in a decrease of p-Akt (Ser473), p-mTOR (Ser2448), p-GSK3β (Ser9), and cyclin D1 protein levels (Fig. 5G). Also, together with Akt inhibitor MK2206 treatment, the enhancement signals of p-Akt (Ser473), p-GSK3β (Ser9), and cyclin D1 in Huh7 and PLC/PRF-5 cells induced by hCAP18/LL-37 were decreased significantly (*p* < 0.001; Fig. 5H). Functional experiments revealed that after blocking Akt using inhibitors, both the enhanced pro-proliferative effect and colony formation ability induced by hCAP18/LL-37 overexpression were reduced greatly (Fig. 5I, J). Taken together, these data suggest that hCAP18/LL-37 expression induced pro-survival and pro-proliferation effects by activating the EGFR-HER2/Akt signaling pathway.

### LL-37 promotes HCC xenograft tumor growth

**Fig. 6:**
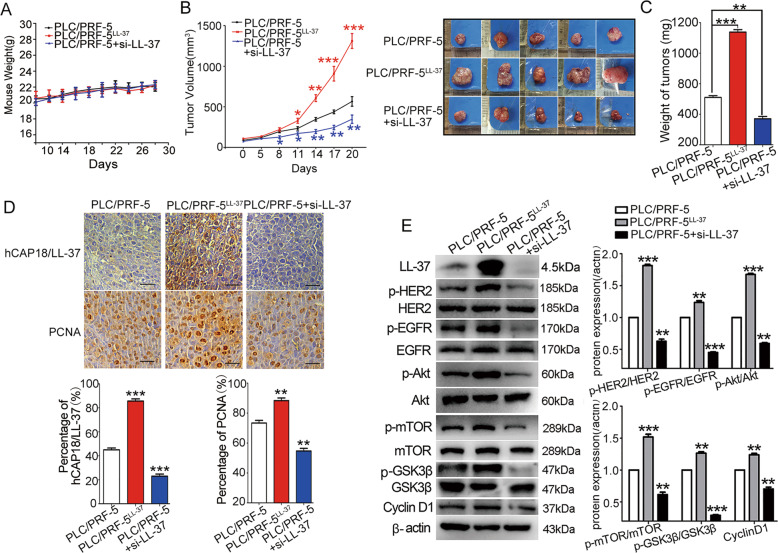
LL-37 expression promotes PLC/PRF-5 xenograft tumor growth. PLC/PRF-5 and PLC/PRF-5^LL-37^ cells were subcutaneously injected into nude mice (4–6 weeks old) to form xenograft mouse models and were assigned into three groups: PLC/PRF-5 xenograft mouse group (control group), PLC/PRF-5^LL-37^ xenograft mouse group, and PLC/PRF-5 xenograft mouse group treated with si-LL-37. Mouse weight (**A**) and tumor volumes (**B**) were determined every 2 days and tumor growth curves over time was illustrated. **B** Mice were sacrificed on day 28 and tumor weight (**C**) was measured. **D** Paraffin sections were prepared for immunohistochemical staining using anti-hCAP18/LL-37 and anti-PCNA antibodies. Representative images from each group are shown. Scale bars, 50 μm. **E** Tumor tissues were crushed, lysed with RIPA lysis buffer, and supernatants were collected for western blotting using the indicated antibodies. Error bars represent the mean ± standard deviation, *n* = 5. *p < 0.05, **p < 0.01, and ***p < 0.001.

To investigate the effect of LL-37 on xenograft tumor growth in vivo, an HCC tumor model was established via subcutaneous injection of PLC/PRF-5 or stable LL-37-overexpressing PLC/PRF-5^LL-37^ cells into BALB/c nude mice. si-LL-37 was used to knock down the expression level of LL-37. Results showed that there were no significant differences in weight in the PLC/PRF-5 (control group), PLC/PRF-5^LL-37^, and PLC/PRF-5 xenograft mouse groups treated with si-LL-37 (Fig. 6A). Compared to the PLC/PRF-5 xenograft group, the tumor growth curve revealed that the tumor growth rate (tumor volume) was significantly increased in PLC/PRF-5^LL-37^ group (Fig. 6B). On the 28th day, the tumor volume was increased by more than two-fold in PLC/PRF-5^LL-37^ xenograft tumors (1311 ± 91 vs. 567 ± 59 mm^3^) compared to PLC/PRF-5 xenograft tumors. However, si-LL-37 treatment in PLC/PRF-5 xenograft mice reduced the tumor volume to 348 ± 52 mm^3^, which was significantly smaller than that in the PLC/PRF-5 xenograft group (*p* < 0.01). Accordingly, the tumor weight was significantly increased from 0.52 ± 0.06 g in the PLC/PRF-5 xenograft tumors to 0.93 ± 0.12 g in the PLC/PRF-5^LL-37^ xenograft tumors (*p* < 0.001; Fig. 6C) and decreased to 0.23 ± 0.08 g when silencing LL-37. Furthermore, hCAP18/LL-37 level was identified in different groups using immunohistochemistry analysis. As expected, the highest expression was in the PLC/PRF-5^LL-37^ group and the lowest expression was in the si-LL-37 group (Fig. 6D). Thus, LL-37 expression effectively promoted tumor growth in the HCC xenograft.

Then, PCNA staining was conducted to assess the effect of LL-37 on proliferation in tumor tissue. Results showed that the positive expression of PCNA was correlated with high expression of LL-37, whereas a lower PCNA level was accompanied by low hCAP18/LL-37 expression. Western blot assay further showed that increasing the expression level of hCAP18/LL-37 significantly increased the levels of p-EGFR (Tyr845), p-HER2 (Tyr1248), p-Akt (Ser473), p-mTORC1 (Ser2448), p-GSK3β (Ser9), and cyclin D1 in xenograft tumors, whereas LL-37 silencing significantly decreased the levels of p-EGFR (Tyr845), p-HER2 (Tyr1248), p-Akt (Ser473), p-mTORC1 (Ser2448), p-GSK3β (Ser9), and cyclin D1 (Fig. 6E). Thus, LL-37 expression significantly promotes tumor growth and upregulates the EGFR/HER2/Akt signals in the PLC/PRF-5 xenograft tumor.

### LL-37 suppresses antitumor activity of 1,25(OH)_2_D_3_ in HCC xenograft tumor

**Fig. 7:**
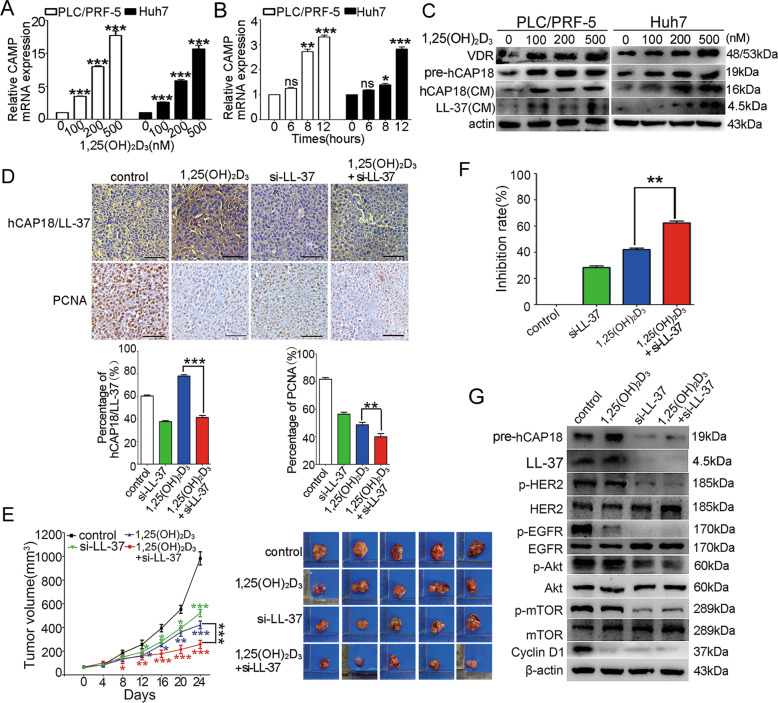
hCAP18/LL-37 silencing improves antitumor activity of 1,25(OH)_2_D_3_ in PLC/PRF-5 xenograft tumor. *CAMP* mRNA levels were detected in PLC/PRF-5 and Huh7 cells by qRT-PCR after different concentrations of 1,25(OH)_2_D_3_ (100, 200, and 500 nM) treatment for 24 h (**A**), or after different time (6 h, 8 h, and 12 h) induced by 200 nM 1,25(OH)_2_D_3_ (**B**). **C** hCAP18 and VDR levels were detected by western blot after 1,25(OH)_2_D_3_ treatment for 48 h. PLC/PRF-5 cells were subcutaneously injected into nude mice (4–6 weeks old) to form xenograft mouse models, which were assigned into PLC/PRF-5 xenograft group (control group) and different treatment groups. On day 37, mice were sacrificed and tumors were carefully excised. **D** Paraffin sections were prepared for immunohistochemical staining using anti-hCAP18/LL-37 and anti-PCNA antibodies. Representative images from each group are shown. Scale bars, 50 μm. **E** Tumors and tumor growth curves over time were illustrated. Difference comparison was conducted between different treatment groups and control group. **F** Comparison of tumor growth inhibition rate between 1,25(OH)_2_D_3_ treatment alone and combination treatment with 1,25(OH)_2_D_3_ and hCAP18/LL-37 silencing. **G** Tumor tissues were crushed and lysed with RIPA lysis buffer, and then the supernatants were collected for western blotting using indicated antibodies. *p < 0.05, **p < 0.01, ***p < 0.001, and ns (no significance).

Given that *CAMP* is an important target gene that is transcriptionally regulated by 1,25(OH)_2_D_3_ in several cell types, it was hypothesized that LL-37 may be a factor that affects the antitumor activity of 1,25(OH)_2_D_3_ in HCC. To identify the effect of 1,25(OH)_2_D_3_ on the expression of hCAP18/LL-37 in cultured HCC cells, 1,25(OH)_2_D_3_ at different concentrations (100 nM, 200 nM, and 500 nM) was added to cells and then *CAMP* expression levels were detected by qRT-PCR analysis. Results showed that the mRNA levels of *CAMP* significantly increased in a concentration-dependent in cultured HCC cells after treatment with 1,25(OH)_2_D_3_ for 24 h (*p* < 0.001; Fig. 7A). Furthermore, the *CAMP* mRNA level increased significantly within 8 h after 200 nM of 1,25(OH)_2_D_3_ treatment, and reached three times the control level after 12 h (Fig. 7B). Further analysis showed that the vitamin D receptor (VDR) had a normal expression in PLC/PRF-5, and Huh7 cells, and the hCAP18 protein levels were increased induced by 1,25(OH)_2_D_3_ in a concentration-dependent manner in all HCC cells (Fig. 7C). Thus, in human HCC cells, hCAP18/LL-37 expression can be significantly induced by the 1,25(OH)_2_D_3_ treatment.

Next, a PLC/PRF-5 xenograft mouse model was constructed to investigate the effect of hCAP18/LL-37 on the antitumor activity of 1,25(OH)_2_D_3_. As expected, the hCAP18/LL-37 levels were significantly elevated in the tumor tissue after 1,25(OH)_2_D_3_ treatment in a PLC/PRF-5 xenograft tumor (*p* < 0.01 Fig. 7D). The 1,25(OH)_2_D_3_ treatment also significantly inhibited tumor growth. Moreover, the tumor growth was further significantly decreased in 1,25(OH)_2_D_3_-treated PLC/PRF-5 xenograft mice when treated together with si-LL-37, compared to 1,25(OH)_2_D_3_ treatment alone (*p* < 0.01, Fig. 7E). However, There was no significant difference in body weight (Fig. S1C). Inhibition rate (tumor weight) for the combination treatment of 1,25(OH)_2_D_3_ with si-LL-37 was significantly higher than that for 1,25(OH)_2_D_3_ treatment alone (*p* < 0.01, Fig. 7F). PCNA staining revealed that combination treatment with 1,25(OH)_2_D_3_ and si-LL-37 showed a more pronounced anti-proliferation effect in HCC tumors than with 1,25(OH)_2_D_3_ alone (Fig. 7D). Accordingly, lower phosphorylation levels of p-HER2 (Tyr1248), p-EGFR (Tyr845), p-Akt (Ser473), p-mTORC1 (Ser2448), and p-GSK3β (Ser9) were observed in tumor tissue after combination treatment with 1,25(OH)_2_D_3_ and si-LL-37, compared to 1,25(OH)_2_D_3_ treatment alone (Fig. 7G). Taken together, these data indicate that 1,25(OH)_2_D_3_ significantly induced the expression of hCAP18/LL-37 in tumor tissue, while hCAP18/LL-37 decreased the antitumor activity of 1,25(OH)_2_D_3_ partly by activating the EGFR/HER2/Akt signaling pathway.

## Discussions

As the only member of the cathelicidin family in humans, hCAP18/LL-37 has several important functions in antimicrobial, wound healing, and immune modulation [23]. This peptide is widely constitutively expressed in various epithelial and innate immune cells, while its induced expression is also present in some non-immune cells, such as keratinocytes and colon epithelial cells [24, 25]. Recently, more studies have revealed that the basal level of hCAP18/LL-37 in different tumor tissues is different. Compared to normal tissues, hCAP18/LL-37 expression levels in most tumor tissues are upregulated, but levels in gastrointestinal cancer (colon and gastric cancers) and hematological malignancies have been reported to be lower than normal [9]. In the present study, the hCAP18/LL-37 level was lower in HCC tissues and cultured cells than in normal liver tissue and cells. It has been reported that the expression of hCAP18 in immune cells could be induced under certain conditions, such as infection, vitamin D, butyrate and short-chain fatty acids [26, 27]. Among these, vitamin D is an important inducer. For example, 1,25(OH)_2_D_3_ or VDR activation can significantly induce hCAP18 expression in acute myeloid leukemia cells [28]. In solid tumors, our present data showed for the first time, that mRNA and protein levels of hCAP18/LL-37 were significantly increased in both cultured HCC cells and xenograft tumors by 1,25(OH)_2_D_3_ treatment. This implies that (1) 1,25(OH)_2_D_3_ treatment might increase the hCAP18/LL-37 level in HCC, and (2) the lower expression of hCAP18/LL-37 in HCC may be associated with a severe deficiency of vitamin D in HCC patients [29]. Therefore, it is very important to reveal the function of LL-37 in HCC and then to elucidate the relationship between vitamin D and hCAP18/LL-37 level in HCC patients.

LL-37 has two different and contradictory effects: promotion or inhibition of tumor growth, mostly depending on the ability of LL-37 to act as a ligand for different membrane receptors whose expression varies based on different cancer cells [9]. LL-37 and its precursor in tissues and body fluids can range from 0.4 to 1 µM. However, concentrations can increase to 4.45 µM during infections in bronchoalveolar fluid [30]. Here, LL-37 promoted cell growth in Huh7, HepG2, and PLC/PRF-5 cells at concentrations ranging from 0.2 to 10 µM and led to necrosis at very high concentration (20 or 40 µM). As a membrane-active antimicrobial peptides (AMPs), LL-37 commonly has a necrotic effect on the cells due to its high concentrations [31]. Many AMPs can lead to necrosis by directly disrupting cancer cell plasma membranes [32]. Consistent with exogenous data, hCAP18/LL-37 overexpression has a tumor-promoting function in HCC both in vitro and in vivo. Thus, although contradictory LL-37 effects are mainly due to the different membrane receptors in different cancer cells, its concentration is another important factor that is important when investigating exogenous LL-37. Here, the HCC cells proliferation and tumor growth were significantly promoted by hCAP18/LL-37 overexpression, while significantly decreased after knocking down of the basal levels of hCAP18/LL-37. Furthermore, the colony formation ability was almost lost after knocking down of the basal levels of *CAMP* in PLC/PRF-5 cells (Fig. 2F). All these suggested that low hCAP18/Ll-37 levels may be enough to promote the survival and proliferation of HCC cells.

There are several receptors that may be associated with tumor-promoting functions of hCAP18/LL-37, such as FPR2, EGFR, and IGFR1 receptor [22, 33]. EGFR overexpression is detected in 68% of HCC cases and is linked to more aggressive tumors with a high proliferation index, intrahepatic metastasis, de-differentiation, and tumor size [34]. HER2 expression is elevated in 30–40% of HCC cases and is involved in the transmission of proliferation and differentiation signals. Researches have revealed that HER2 protein is overexpressed in both HCC cell lines and tumor tissues, and that HER2 expression is related to in vitro and in vivo proliferation and HCC Epithelial-Mesenchymal Transition (EMT) [35]. LL-37 has been reported to be linked to EGFR signaling in a variety of cells, including lung cancer, corneal epithelial, and airway epithelial cells [23, 36]. A functional association between LL-37 and HER2 was identified in breast cancer [37]. Our data demonstrated that both EGFR and HER2 are important targets for hCAP18/LL-37 in HCC, resulting from overexpression of hCAP18/LL-37 significantly activating phosphorylation of both EGFR and HER2 in cultured HCC cells and xenograft tumors. HER2 has a functionless ligand-binding domain and its activation relies on dimerization with other activated ErbB family members. It is possible that hCAP18/LL-37 promotes some ligand binding to EGFR, leading to EGFR/HER2 dimerization and autophosphorylation and then to activation of EGFR/HER2.

The exact mechanism for hCAP18/LL-37 activation of EGFR/HER2 remains unclear. One proposed mode of action is that LL-37-induced GPCR activation stimulates a currently unidentified member of the disintegrin and metalloproteinase family, which subsequently cleaves a membrane-anchored EGFR ligand, such as HB-EGF or transforming growth factor-alpha (TGF-α). Metalloproteinases are responsible for ectodomain shedding of membrane-anchored EGFR ligands, such as TGF-α, amphiregulin, and HB-EGF, which potentially bind to and activate EGFR [36]. Several studies have postulated that LL-37 transactivates EGFR via metalloproteinase-mediated cleavage of membrane-anchored pro-EGFR ligands to release HB-EGF in epithelial cells and keratinocytes, regulating keratinocyte migration or promoting wound healing [22]. Here, we have observed that hCAP18/LL-37 significantly promoted HB-EGF release from membrane-anchored pro-HB-EGF in HCC cells. This implies that inducing the HB-EGF release by hCAP18/LL-37 is an important way to activate EGFR/HER2 in HCC cells, although how hCAP18/LL-37 regulates HB-EGF release is still unknown.

In lung cancer cells, LL-37 upregulates the phosphorylation of EGFR and causes a subsequent activation of the Ras/MAPK cascade [38]. In breast cancer cells, LL-37 increases the phosphorylation level of HER2 and then activates MAPK signaling [37]. The present study found several tumor-promoting pathways including the PI3K/Akt, MAPK, and JAK/STAT pathways, related to the functions of LL-37 overexpression in HCC cells using KEGG analysis. Importantly, the PI3K/Akt signaling pathway is the most obvious upregulated signaling pathway induced by LL-37 expression, followed by the MAPK pathway. PI3K/Akt/mTOR signaling pathway is activated in 30–50% of HCC patients and plays a significant function in cell growth, survival regulation, metabolism, and anti-apoptosis in HCC [5]. hCAP18/LL-37 overexpression significantly phosphorylated Akt in PLC/PRF-5 and Huh7 cells, while blocking EGFR/HER2 by inhibitors resulted in a significant decrease in the Akt signal. Furthermore, Akt inhibition with MK2206 repressed the activation of mTORC1, GSK3β, and pro-proliferative effect induced by hCAP18/LL-37. In addition to mTORC1, GSK3β is another downstream target of Akt, which can be directly phosphorylated at Ser9 by Akt in HCC cells. Therefore, the PI3K/Akt/GSK3β/cyclin D1 pathway is another signaling pathway induced by LL-37. As a critical oncogenic protein, Akt regulates cell survival, proliferation, growth, apoptosis, and glycogen metabolism [39]. A previous report has revealed that as FPR2 agonist, LL-37 triggers FPR2 and subsequent ROS/MAPK/NF-kB signaling, upregulates M-CSF/MCP-1 expression, leads to M2b- or M2d-like polarization, and exacerbates HCC invasion in vitro and in vivo [40]. Our current data revealed several tumor-promoting signals in HCC cells, such as the PI3K/Akt and MAPK pathways that were stimulated by LL-37. Thus, multiple mechanisms might be involved in the HCC tumor growth induced by LL-37. Compared to LL-37 silencing, tumor volume and weight increased about three-fold in LL-37-overexpressing HCC xenograft mice, indicating that the pro-tumor activity in vivo was stronger than that in vitro. We speculate that there are multiple carcinogenesis mechanisms in LL-37 in vivo. Indeed, LL-37 overexpression promoted pulmonary metastasis in xenograft tumor mice (data not shown).

In recent years, vitamin D supplementation has attracted more and more attention in various malignancy therapies (www.clinicaltrials.gov). Preclinical studies in animal models have demonstrated that vitamin D treatment may inhibit the development in several types of cancer, by reinforcing the concept that vitamin D plays a crucial role in carcinogenesis [41]. However, clinical studies on 1,25(OH)_2_D_3_ or its analogs alone do not always show improvements in tumor progression and mortality risk, particularly for HCC treatment, indicating that the anticancer activity of 1,25(OH)_2_D_3_ in HCC may be affected by many unknown factors. Specifically, 1,25(OH)_2_D_3_ activates VDR, which in turn modulates the expression of nearly 200 primary vitamin D target genes in human monocytes [42]. It has been reported that hCAP18/LL-37 is one of the most relevant vitamin D target genes in the immune cells [19]. The present study found that 1,25(OH)_2_D_3_ significantly induced the expression of hCAP18/LL-37 in both HCC cells and xenograft tumor tissue. More interestingly, 1,25(OH)_2_D_3_ treatment together with interfering the expression of hCAP18/LL-37 significantly enhanced the antitumor activity of 1,25(OH)_2_D_3_ in the HCC xenograft tumor. As mentioned above, LL-37 had pro-tumoural activities in HCC via activating multiple oncogenic signals. This implied that hCAP18/LL-37 may be an important factor that suppresses the therapeutic benefit of 1,25(OH)_2_D_3_ in HCC tumors. Macrophages are an important component of the HCC microenvironment. It has been reported that *CAMP* is strongly expressed in macrophages induced by 1,25(OH)_2_D_3_ [10]. Thus, whether the expression of hCAP18/LL-37 level induced by 1,25(OH)_2_D_3_ in macrophages in HCC microenvironment has a stronger suppressive effect on the antitumor activity of 1,25(OH)_2_D_3_ in vivo requires further investigation urgently. The related research work is being carried out in our lab at present.

In conclusion, the present study demonstrated that human cathelicidin hCAP18/LL-37 has a cancer-promoting effect in HCC both in vitro and in vivo. Mechanistically, hCAP18/LL-37 promotes the release of HB-EGF, upregulates the phosphorylation of EGFR/HER2, activates the PI3K/Akt signaling pathway, and promotes HCC cells proliferation and tumor growth. More interestingly, hCAP18/LL-37 was also an important factor that suppressed the antitumor activity of 1,25(OH)_2_D_3_ in the HCC xenograft tumor. Thus, 1,25(OH)_2_D_3_ treatment together with targeting hCAP18/LL-37 expression silencing may be a potential strategy to improve the anticancer activity of 1,25(OH)_2_D_3_ in HCC treatment.

## Materials and methods

### Cell lines, tissue samples, and mice

Human HCC cell lines Huh7, PLC/PRF-5, and HepG2 and normal human hepatic L02 cells were purchased from Shanghai Institute of Cell Biology, Chinese Academy of Science. Huh7, PLC/PRF-5, and HepG2 cells were maintained in DMEM, while L02 cells were maintained in RPMI-1640 medium supplemented with 10% fetal bovine serum, penicillin (100 U/mL), and streptomycin (100 mg/mL) at 37 °C in 5% CO_2_ in a humidified atmosphere. Human HCC tissue (60 cases) and corresponding adjacent normal tissue (60 cases) chips were purchased from Shanghai Outdo Biotech (Shanghai, China). Informed consent was obtained from all patients, and the study was approved by the ethics committee of Nanjing Normal University. Male BALB/c nude mice (4–6 weeks old, 18.0 ± 2.0 g) were purchased from the Model Animal Research Institute of Nanjing University. Male BALB/c nude mice were maintained in specific pathogen free (SPF) conditions in accordance with the guidelines of the laboratory animal ethics committee of Nanjing Normal University.

### Reagents, antibodies, and plasmids

LL-37 was synthesized by Synpeptide Inc (Nanjing, China). RPMI-1640 and DMEM were purchased from Thermo Fisher Scientific (Waltham, MA, USA), fetal bovine serum (FBS) from Capricorn scientific (Hessen, Germany). HiTrans^TM^ LipoPlus Reagent was purchased from Jiangsu Synthgene Biotechnology Co., Ltd. (Nanjing, China). Entranster-in vivo DNA transfection reagent was purchased from Engreen Biosystem Co, Ltd. (Beijing, China). Penicillin and streptomycin were purchased from Solarbio (Beijing, China). 1,25(OH)_2_D_3_, MK2206, and Neratinib were purchased from MedChemExpress (Monmouth Junction, NJ, USA). Hoechst 33342/PI was purchased from KeyGEN Biotech (Nanjing, China). Antibodies against hCAP18/LL-37, VDR, EGFR, p-EGFR (Tyr845), p-HER2 (Tyr1248), p-Akt (Ser473), GSK3β, p-GSK3β (Ser9) were purchased from Santa Cruz (CA, USA). Antibodies against mTOR and p-mTOR were purchased from Abways (Shanghai, China). Antibodies against HER2, Akt, cyclin D1, and PCNA were purchased from Cell Signaling Technology (MA, USA). Antibodies against HB-EGF, β-actin, anti-rabbit, and anti-mouse secondary antibodies were purchased from Abclonal (Wuhan, China). TRIzol reagent, Taq Master Mix, HiScriptIII RT SuperMix for qPCR (+gDNA wiper), AceQ qPCR SYBR Green Master Mix (High ROX Premixed), and CCK-8 were acquired from Vazyme (Nanjing, China). DAB was acquired from Beyotime (Nanjing, China). pRNAT-U6.1-GFP and pcDNA3.0-Flag expression vector was kept in our laboratory. The sequence (5'-GTCCAGAGAATCAAGGATT-3') specifically targets the encoding LL-37 of *CAMP* gene (si-LL-37) and negative control siRNA (scrambled siRNA, si-control) were synthesized by General Biol (Anhui, China), respectively. All other reagents were analytical grade reagents and produced in China.

### MTT assay

Cells (Huh7, PLC/PRF-5, HepG2, and L02 cells) were seeded in 96-well plates at a density of 1 × 10^5^ cells/mL in medium, and then treated with different concentrations of LL-37 (0, 0.2, 0.4, 0.6, 0.8, 1, 2, 4, 6, 8, 10, 20, and 40 μM) for 24 h. After treatment, 20 μL of 5 mg/mL MTT was added, and the plates were incubated at 37 °C for 4–8 h. And then 150 μL DMSO was added to dissolve formazan. Finally, the absorbance at 570 nm (test wavelength) and 630 nm (reference wavelength) was detected using a Synergy^TM2^ Multifunction Microplate Reader. The viability percentage was calculated as: (OD_LL-37_-OD_CM_)/(OD_PBS_-OD_CM_) × 100%. Data were displayed in the figures as mean SEM for 4–6 independent experiments.

### Plasmid construction, transfection, and stable LL-37-overexpressing cell generation

Full-length pre-hCAP18 (pre-hCAP18) and LL-37 coding sequences (*GenBank accession no. 820*) were amplified by qRT-PCR from mRNA of L02 cells using Oligo dT_23_ that incorporated *Bam HI* and *Xbal I* restriction sites. *CAMP* cDNA and DNA coding LL-37 fragment were inserted into pcDNA3.0 via *Bam HI/XbalI*restriction sites to generate the pcDNA/hCAP18 and pcDNA/LL-37 constructs, respectively. Constructs were confirmed by sequencing. The primers were shown in the supplementary materials (Supplementary Table S1).

Transient transfection was performed using HiTrans^TM^ LipoPlus reagent according to the manufacturer’s protocol. Briefly, cells (3 × 10^5^ cells/mL) were seeded into six-well plates. After incubation for 12 h, 2 μg of pcDNA3.0, pcDNA/hCAP18, pcDNA/LL-37, si-control, and si-LL-37 were added, respectively. A GFP expression vector pRNAT-U6.1-GFP was co-transfected to monitor transfection efficiency. Only cells with transfection efficiency over 90% were used for followed up study (Supplementary, Fig. S1B).

To establish stable LL-37-expressing PLC/PRF-5LL-37 cells, G418 (800 μg/mL) was added to the cell medium to obtain G418-resistant cell lines after 24 h of transient transfection. During the screening, the culture medium containing G418 was changed every 3–5 days. Selection of recombinant transfectants was performed for at least 30 days. Finally, the constructed stable PLC/PRF-5^LL-37^ cells were identified by qRT-PCR and western blotting.

### Neratinib or MK2206 treatment

To assess the effect of neratinib or MK2206, 3 μM neratinib or 5 μM MK2206 was added to the culture medium after transient transfection for 24 h. After incubation for another 24 h, cells were collected for further analysis.

### CCK-8 assay

CCK-8 assay was performed using CCK-8 kit following the manufacturer’s recommendations. Briefly, cells (Huh7, PLC/PRF-5) were seeded in six-well plates at a density of 3 × 10^5^ cells/mL. After cells adhesion, transfection assay was conducted. Approximately 1 × 10^3^ transfected cells were seeded in 96-well plates for more than 24 h. Then, 10 μL of CCK-8 solution was added to each well at different times and incubated at 37 °C for 2 h. Microplate Autoreader was used to measure optical density at 450 nm. Three independent experiments were conducted, and each experiment was repeated 4–6 times.

### Colony formation assays

Cells (500 or 1000 cells/well) were seeded in 12-well plates and cultured in DMEM at 37 °C. The cells were fixed with 4% paraformaldehyde and stained with crystal violet (0.1%) for about 10 days. Visible colonies (more than 50 cells) were counted and photographed under a light microscope. Each experiment was repeated at least three times.

### 5-Ethynyl-20-deoxyuridine (EdU) incorporation assay

Cell proliferation assays were performed using the BeyoClick™ EdU-555 kit per the manufacturer’s instructions. After transfection for 48 h, cells were treated with EdU (20 μM) for 2 h at 37 °C, fixed with 4% paraformaldehyde for 15 min, and permeabilized with 0.2% Triton X-100 for 15 min. Cells were then incubated in an EdU staining cocktail at room temperature in the dark for 30 min. After washing with PBS, cell nuclei were stained with Hoechst 33342 (10 μg/mL) for 15 min. More than five images of each well were captured using a fluorescence microscope equipped with a filter for Ex/Em = 555/565 nm. EdU-positive and total cells were counted within each field.

### Cell cycle assay

For cell cycle analysis, cells were harvested and fixed in cold 70% ethanol at 4 °C overnight. Then, cells were stained with propidium iodide (PI) and RNAase solution for 30 min at 37 °C in the dark. Cell distribution across the cell cycle was analyzed with a FACSVerse flow cytometer with an excitation wavelength of 488 nm. Flowjo ten analysis software was used for cellular DNA content analysis.

### Real-time polymerase chain reaction (qRT-PCR)

Cells (2 × 10^5^/mL) were seeded into 6-well plates and treated with 500 nM of 1,25(OH)_2_D_3_ for 6 h, 12 h, and 24 h. RNA was extracted from cells using Trizol reagent and reverse transcribed with SuperScript II reverse transcriptase to generate complementary DNA (cDNA). SYBR Green PCR Kit was used for qRT- PCR with β-actin as the internal control. The reaction was performed using a StepOne Plus Real-Time PCR system. Briefly, 20 μL-reactions (10 μL of SYBR Green qPCR Master Mix, 50 ng of total RNA, and 0.4 μL of forward and reverse primers) was subjected to one cycle of 95 °C for 10 min and then 40 cycles of 95 °C for 5 s, 60 °C for 60 s, and 72 °C for 1 min. The primers were shown in the supplementary materials (Supplementary Table S2). Relative gene expression levels were calculated based on the 2^−ΔΔCt^ method.

### GEPIA an UALCAN analysis

GEPIA (http://gepia.cancer-pku.cn/) is an interactive web server for cancer and normal gene expression profiling and interactive analyses based on 9736 tumor samples across 33 cancer types and 726 adjacent normal tissues from the TCGA (The Cancer Genome Atlas), and over 8000 normal samples from the GTEx (Genotype-Tissue Expression) databases. The GEPIA database was used to compare mRNA levels of *CAMP* in HCC between TCGA and GTEx databases. Meanwhile, UALCAN (http://ualcan.path.uab.edu) uses TCGA level 3 RNA-seq and clinical data from 31 cancer types as an interactive web resource. In this study, UALCAN was used to analyze the mRNA expressions of *CAMP* in HCC between normal tissues and normal weight HCC tissues.

### Transcriptome sequencing analyses

Total RNA for PLC/PRF-5 and PLC/PRF-5^LL-37^ cells was extracted using TRIzol Reagent according to the manufacturer’s instructions. The RNA samples were sent to Shanghai Majorbio Bio-pharm Technology Co., Ltd (Shanghai, China) for whole transcriptome sequencing. Briefly, 1 μg of total RNA with RIN value of >6.5 was used for library preparation. mRNA was isolated using Poly(A) mRNA Magnetic Isolation Module kit. Then, cDNA was synthesized using ProtoScript II Reverse Transcriptase and Second Strand Synthesis Enzyme mix. Fragments of ~420 bp in size (with an approximate insert size of 300 bp) were selected, amplified, and validated using Qsep100 (Bioptic, Taiwan, China). Libraries were then sequenced on an Illumina HiSeq instrument. The data were analyzed using the free online Majorbio Cloud Platform (www.majorbio.com).

### Bioinformatics analysis

Differential expression analysis was conducted by the DESeq2 Bioconductor package, a model based on the negative binomial distribution. The estimates of dispersion and logarithmic fold changes incorporate prior data-driven distributions, where *p* < 0.05 detects differential expression. Gene ontology and Kyoto Encyclopedia of Genes and Genomes (KEGG) pathway enrichment analyses of differentially expressed genes were evaluated using GOSeq (v1.34.1). In-house scripts were used to enrich significant differentially expressed genes in the KEGG pathways. In addition, changes in the expression of differentially expressed genes were greater than two-fold and *p* < 0.05 was used to screen and count downregulation of significant differentially expressed genes to generate volcano plots.

### Western blotting

Cells were collected and the lysates were separated on 8–15% SDS-PAGE gels and transferred to PVDF membranes. The membranes were blocked using 5% non-fat milk. Then, the PVDF membranes were incubated with primary antibodies (1:1000) at 4 °C overnight, followed by incubation with an HRP-conjugated anti-mouse or anti-rabbit secondary antibody separately. Finally, the bands were detected using an Odyssey infrared imaging system and analyzed with ImageJ densitometry analysis software. To determine hCAP18, LL-37, HB-EGF in the culture medium, culture supernatant samples were collected and concentrated with methanol and trichloromethane to conduct western blotting. After weighing xenograft tumor tissue samples, RIPA lysis buffer (400 μL/30 mg) was added to prepare tissue homogenates using a tissue homogenizer. Lysates were centrifuged to collect the supernatant, followed by western blotting as described above.

### In vivo experiments

The xenografted animal assays were carried out in two batches. To assess the effect of LL-37 on the tumor growth, male BALB/c nude mice (4–6 weeks old, 18.0 ± 2.0 g) were injected subcutaneously into the right axillary region with 6 × 10^6^ of PLC/PRF-5 or PLC/PRF-5^LL-37^ cells. When the mean tumor volume reached 50–100 mm^3^, the nude mice were randomly divided into three groups (5–6 mice per group), including (1) PLC/PRF-5, (2) PLC/PRF-5^LL-37^, and (3) PLC/PRF-5 mouse groups injected with si-LL-37 (5 µg per mouse) every 3 days. In all the in vivo experiments, si-LL-37 was the modified si-LL-37 with 2'-O-Methylbase in order to extend the half-life in animal body. To assess the effect of LL-37 on the anticancer activity of 1,25(OH)_2_D_3_, male BALB/c nude mice (4–6 week old, 18.0 ± 2.0 g) were injected subcutaneously into the right axillary region with 6 × 10^6^ of PLC/PRF-5. When the mean tumor volume reached 50–100 mm^3^, the nude mice were randomly divided into four groups (5–6 mice per group), including (1) PLC/PRF-5 group, (2) PLC/PRF-5 mouse groups intraperitoneal injected with 1,25(OH)_2_D_3_ at a dose of 0.5 μg/kg/per day, (3) PLC/PRF-5 mouse groups injected with si-LL-37 (5 µg per mouse) every 5 days, and (4) PLC/PRF-5 mouse groups intraperitoneal injected with 1,25(OH)_2_D_3_ at a dose of 0.5 μg/kg/per day and si-LL-37 (5 µg per mouse) every 5 days. Here, noncalcaemic dose of 1,25(OH)_2_D_3_ was used on the basis of data as described previously [43]. Tumor volume and weight of each mouse were recorded. Tumor volume was calculated using the formula *V* = ab^2^/2, where “a” and “b” are tumor dimensions at the longest and widest points, respectively. The mice were housed for 4 weeks and sacrificed by isoflurane inhalation followed by cervical dislocation. The tumors were excised and weighed. The rate of tumor growth inhibition was calculated from the tumor weight (TW, g) as tumor growth inhibition rate = (TW_control_-TW_Treatment_)/TW_control _× 100%. The animal protocols complied with ethical regulations and were approved by the Animal Welfare Committee of Nanjing Normal University.

### Immunohistochemistry assay

Xenograft tumors were fixed in 4% paraformaldehyde for 24 h followed by gradient alcoholic dehydration. Then were cleaned, embedded in paraffin, and cut into 5-μm-thick sections. For antigen retrieval, the sections were boiled (100 °C, 20 min) in sodium citrate antigen retrieval solution, cooled naturally for more than 20 min, and washed in PBS for 5 min three times. The blocking agent was then added for 1 h. After washing with PBS, the sections were incubated with primary anti-LL-37 (1:250 dilution) or anti-PCNA (1:250 dilution) overnight at 4 °C. HRP-conjugated goat anti-rabbit IgG secondary antibody was then added. After washing with PBS three times, proteins were then detected using a DAB kit, after which a counterstain with hematoxylin was performed. Finally, after dehydration with gradient ethanol and clearance with xylene, the images were obtained using an Olympus IX51 fluorescence microscope. Percentage of positive cells or the percentage of fluorescence intensity were performed using ImageJ software (https://imagej.nih.gov/ij/).

### Statistical analysis

Values were expressed as the mean ± SEM from 3–6 independent experiments. Two-tailed Student’s *t*-test and one-way ANOVA with Dunnett’s multiple comparison test were used to determine the significance of differences. For all cases, *p* < 0.05 was considered statistically significant. Statistical analysis was assessed using Statistical Package for the Social Sciences (SPSS/PC 20.0, Chicago, IL, USA). *p < 0.05, **p < 0.01, ***p < 0.001.

## Supplementary information

Supplementary figure legends

Supplementary Table S1

Supplementary Table S2

Figure S1

## Data Availability

The data that support the findings of this study are available from the corresponding author upon reasonable request.
