# Supplementary material for: Cathelicidin hCAP18/LL-37 promotes cell proliferation and suppresses antitumor activity of 1,25(OH)2D3 in hepatocellular carcinoma
Source: Cell Death Discov. 2022 Jan 17;8:27. doi: 10.1038/s41420-022-00816-w (PMC8763942; doi:10.1038/s41420-022-00816-w)
Supplement: Supplementary file 1 — Supplementary figure legends [file 41420_2022_816_MOESM1_ESM.docx]

**Spplementary Figure Legends**

**Spplementary Figure S1.** A. Hoechst 33342/PI staining was used to measured the effects of LL-37 on cell apoptosis and necrosis. When confluency reached 80%, approximately 2 × 10^5^ PLC/PRF-5, Huh7, and HepG2 cells were seeded in six-well plates and treated with LL-37 (20, 40 μM) for 24 h. Then, apoptotic nuclei were stained with Hoechst 33342 (v/v at 1:200) and PI (v/v at 1:200) at 37ºC for 30 min in the dark, followed by washing three times with PBS. The images were obtained using fluorescence microscopy. B. The transfection efficiency was assessed with a pRNAT-U6.1-GFP plasmid transfection. In the same experimental group (pcDNA/hCAP18, pcDNA/LL-37, sh-control, and sh-LL-37), GFP plasmid was also transfected in cells, and the transfection efficiency was detected by intensity of GFP expression using fluorescent microscopy in 24 hours posttransfection. C. Mouse weight growth curves over time. PLC/PRF-5 cells were subcutaneously injected into nude mice (4–6 weeks old) to form xenograft mouse models, which were assigned into PLC/PRF-5 xenograft group (control group) and different treatment groups. Mouse weight growth curves over time. Difference comparison was conducted between different treatment groups and control group.
