# Supplementary material for: Cathelicidin hCAP18/LL-37 promotes cell proliferation and suppresses antitumor activity of 1,25(OH)2D3 in hepatocellular carcinoma
Source: Cell Death Discov. 2022 Jan 17;8:27. doi: 10.1038/s41420-022-00816-w (PMC8763942; doi:10.1038/s41420-022-00816-w)
Supplement: Supplementary file 2 — Supplementary Table S1 [file 41420_2022_816_MOESM2_ESM.docx]

**Supplementary table S1. Primers used in construction of pcDNA/hCAP18 and pcDNA/LL-37 plasmid experiments.**

| Gene Name | Primers | Nucleotide Sequence (5’ to 3’) | amplified product lengths |
| --- | --- | --- | --- |
| pcDNA/hCAP18 | F | CGGGATCC ATGAAGACCCAAAGGGATGG | 513bp |
|  | R | GCTCTAGA CTAGGACTCTGTCCTGGGTA |  |
| pcDNA/LL-37 | F | CGGGATCC CTGCTGGGTGATTTCTTCCG | 114bp |
|  | R | GCTCTAGA GGACTCTGTCCTGGGTACAA |  |
