# Supplementary material for: Cathelicidin hCAP18/LL-37 promotes cell proliferation and suppresses antitumor activity of 1,25(OH)2D3 in hepatocellular carcinoma
Source: Cell Death Discov. 2022 Jan 17;8:27. doi: 10.1038/s41420-022-00816-w (PMC8763942; doi:10.1038/s41420-022-00816-w)
Supplement: Supplementary file 3 — Supplementary Table S2 [file 41420_2022_816_MOESM3_ESM.docx]

**Supplementary table S2.** **Oligonucleotide sequences of forward and reverse primers for quantitative real-time PCR (qRT-PCR).**

| Gene Name | Primers | Nucleotide Sequence (5’ to 3’) | Tm (°C) | Species |
| --- | --- | --- | --- | --- |
| *CAMP* | F | CGACACAGCAGTCACCAGAGGA | 60 | Homo |
|  | R | GAAATCACCCAGCAGGGCAAATC | 60 | Homo |
| *β-actin* | F | GAGCTACGAGCTGCCTGACG | 60 | Homo |
|  | R | CCTAGAAGCATTTGCGGTGG | 60 | Homo |
