# Supplementary figures and images for: Cathelicidin hCAP18/LL-37 promotes cell proliferation and suppresses antitumor activity of 1,25(OH)2D3 in hepatocellular carcinoma
Source: Cell Death Discov. 2022 Jan 17;8:27. doi: 10.1038/s41420-022-00816-w (PMC8763942; doi:10.1038/s41420-022-00816-w)

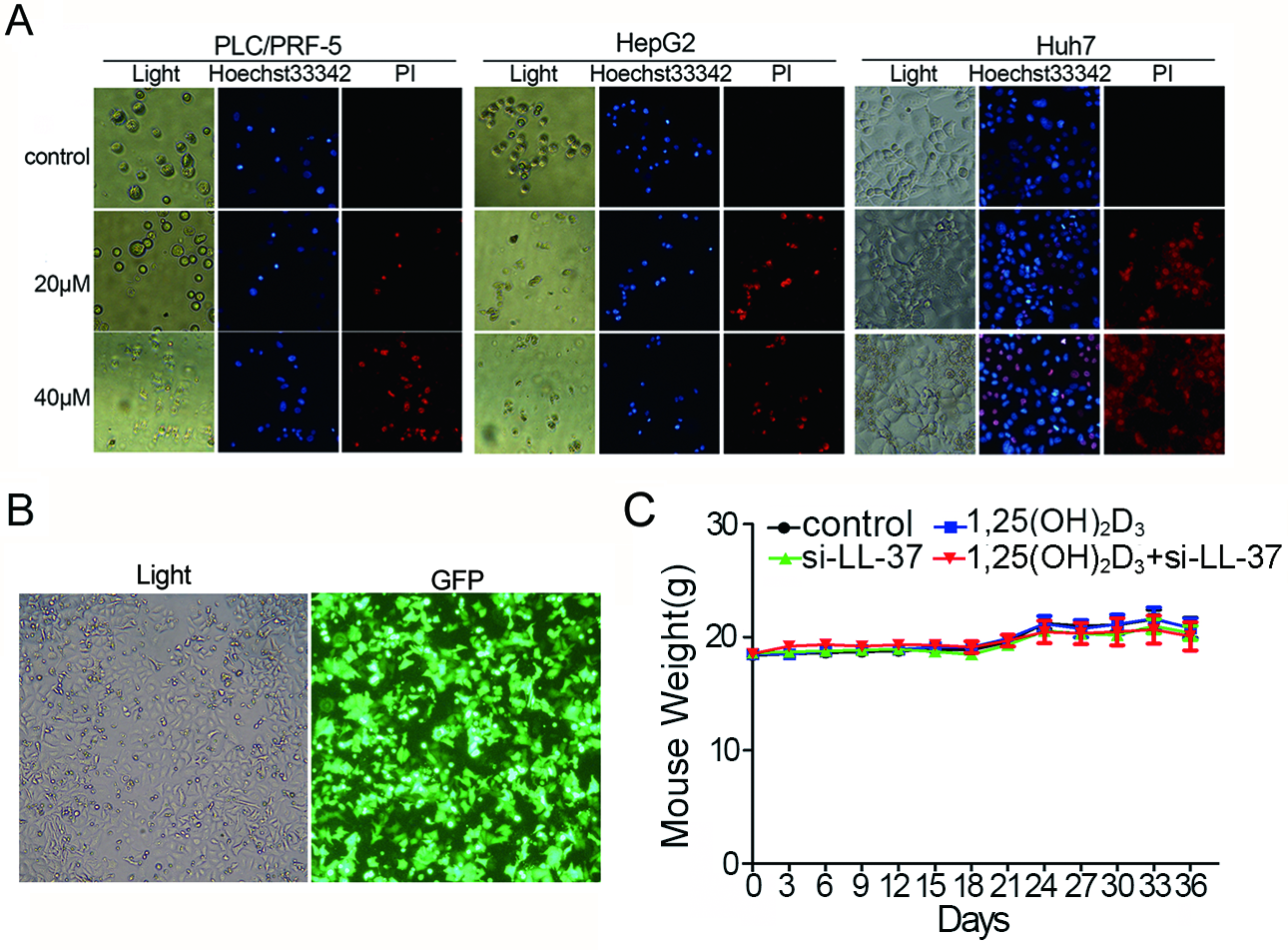

Supplement: Supplementary file 4 — Figure S1 [file 41420_2022_816_MOESM4_ESM.tif]
